# Characterization of microbial community and antibiotic resistome in intra urban water, Wenzhou China

**DOI:** 10.3389/fmicb.2023.1169476

**Published:** 2023-06-15

**Authors:** Sheng Ye, Shengkai Li, Chenjun Su, Zhuqing Shi, Heng Li, Jiawen Hong, Shengke Wang, Jingyan Zhao, Weiji Zheng, Shixuan Dong, Shuhan Ye, Yongliang Lou, Zhemin Zhou, Jimei Du

**Affiliations:** ^1^Wenzhou Key Laboratory of Sanitary Microbiology, Department of Microbiology and Immunology, School of Laboratory Medicine, Wenzhou Medical University, Wenzhou, China; ^2^Pasteurien College, Suzhou Medical College, Soochow University, Suzhou, Jiangsu, China; ^3^Taizhou Hospital of Zhejiang Province, Taizhou, China

**Keywords:** waterborne pathogen, antibiotic resistance, metagenomic analysis, public health, environment

## Abstract

The present study investigated the water quality index, microbial composition and antimicrobial resistance genes in urban water habitats. Combined chemicals testing, metagenomic analyses and qualitative PCR (qPCR) were conducted on 20 locations, including rivers from hospital surrounds (*n* = 7), community surrounds (*n* = 7), and natural wetlands (*n* = 6). Results showed that the indexes of total nitrogen, phosphorus, and ammonia nitrogen of hospital waters were 2–3 folds high than that of water from wetlands. Bioinformatics analysis revealed a total of 1,594 bacterial species from 479 genera from the three groups of water samples. The hospital-related samples had the greatest number of unique genera, followed by those from wetlands and communities. The hospital-related samples contained a large number of bacteria associated with the gut microbiome, including *Alistipes*, *Prevotella*, *Klebsiella*, *Escherichia*, *Bacteroides*, and *Faecalibacterium*, which were all significantly enriched compared to samples from the wetlands. Nevertheless, the wetland waters enriched bacteria from *Nanopelagicus*, *Mycolicibacterium* and *Gemmatimonas*, which are typically associated with aquatic environments. The presence of antimicrobial resistance genes (ARGs) that were associated with different species origins in each water sample was observed. The majority of ARGs from hospital-related samples were carried by bacteria from *Acinetobacter*, *Aeromonas* and various genera from *Enterobacteriaceae*, which each was associated with multiple ARGs. In contrast, the ARGs that were exclusively in samples from communities and wetlands were carried by species that encoded only 1 to 2 ARGs each and were not normally associated with human infections. The qPCR showed that water samples of hospital surrounds had higher concentrations of *intI1* and antimicrobial resistance genes such as *tetA, ermA, ermB, qnrB, sul1, sul2* and other beta-lactam genes. Further genes of functional metabolism reported that the enrichment of genes associated with the degradation/utilization of nitrate and organic phosphodiester were detected in water samples around hospitals and communities compared to those from wetlands. Finally, correlations between the water quality indicators and the number of ARGs were evaluated. The presence of total nitrogen, phosphorus, and ammonia nitrogen were significantly correlated with the presence of *ermA* and *sul1*. Furthermore, *intI1* exhibited a significant correlation with *ermB*, *sul1*, and *bla*_SHV_, indicating a prevalence of ARGs in urban water environments might be due to the integron *intI1*’s diffusion-promoting effect. However, the high abundance of ARGs was limited to the waters around the hospital, and we did not observe the geographical transfer of ARGs along with the river flow. This may be related to water purifying capacity of natural riverine wetlands. Taken together, continued surveillance is required to assess the risk of bacterial horizontal transmission and its potential impact on public health in the current region.

## Highlights

- Water samples collected near hospitals contained greater concentrations of nitrogen, phosphate, and antimicrobial-resistant bacteria.
- Pathogenic and MDR bacteria were detected in urban surface water, and samples from hospital surrounds had a significantly higher abundance and diversity of pathogenic bacteria than the other two’s.
- Metagenomic analysis revealed that both bacterial chromosomes and plasmids harbored various kinds of ARGs in urban water habitats.
- The presence of MDR bacteria and ARGs in urban waterways highlights the importance of further surveillance of antimicrobial resistance in the context of one health.

## 1. Introduction

Antimicrobial resistance genes (ARGs) are emerging environmental contaminants and are frequently found in groundwater, surface water, healthcare facilities, and other aquatic habitats (Zainab et al., 2020). These genes can be absorbed by plants in agroecosystems through the reuse of wastewater, posing exposure threats to humans and animals. Recent studies have reported the emergence of extended-spectrum beta-lactamase-producing (ESBL) bacteria harboring multi-drug resistance (MDR) to beta-lactams, tetracyclines, and aminoglycosides in surface water and wastewater (Haberecht et al., 2019; Gekenidis et al., 2020; Johnson et al., 2020). Considering the environmental residues of antimicrobials in the treatment of human and livestock infections caused by excrement, further antimicrobial-resistant surveillance and a deeper knowledge of how resistance spreads in environmental waterways are warranted under the framework of One Health.

MDR strains harboring divergent ARGs and virulence factor genes (VFs) are pathogenic and may increase the risk of waterborne diseases (Gekenidis et al., 2020). Previous studies revealed that the mobile elements encoding multi-drug resistances jumped its hosts among Enterobacteriaceae via horizontal gene transfer (HGT), leading to rapid dissemination of resistance genes including *bla*_CTX-M_, *bla*_OXA_, *bla*_SHV_, *bla*_TEM_, *bla*_KPC_, and other genes (Dirar et al., 2020; Che et al., 2021). As HGT of ARGs into human pathogen repertoires becomes a growing concern, monitoring multi-drug resistant bacteria in the environment is increasingly important. Notably, class I integron was tightly associated with the transfer of many ARGs and therefore had been used as a proxy for the level of antimicrobial resistance genes in the environment (Liao and Chen, 2018). Therefore, additional research into the associations between ARGs and VFs and mobile elements of MDR strains in water bodies, as well as an examination of the potential role of mobile elements in the transmission of waterborne illnesses, is extremely important for public health.

The present study was a region-specific research conducted in Wenzhou, Zhejiang province. Wenzhou is located on the southeast coast of China, between latitudes 27°94’N to 28°03’N and longitudes 120°59′E to 120°74′E. Wenzhou has a resident population of 2.15 million (accessed on May 2023), and there are more than 195 waterways across the city. Previous researches had revealed the bacterial communities in estuary sediments in the south of Zhejiang Province, suggesting that chemical pollution might destroy the natural variability of the current estuary ecosystems (Lu et al., 2016). Additionally, Li et al. reported the co-occurrence of crAssphage and ARGs in agricultural soils in Southeast China (Li W. et al., 2021). The frequency and abundance of ARGs abundance increased together with the continued feces contamination in the soil. However, the microbiome associated with accumulation of the antimicrobial resistance in water habitats has been rarely studied, let alone how environmental factors shape their relationship. Therefore, it is necessary to illustrate the associations between the microbial composition, distribution of pathogenic bacteria and ARGs in urban waters.

Additionally, the water quality, i.e., nutrients and chemical pollution, is an important environmental factor for microbial community composition (Petersen et al., 2005). In addition, previous studies revealed that the contents of total nitrogen, ammonia nitrogen and total phosphorus were significantly correlated with the abundance of ARGs such as *sul1, sul2, tetA, tetB, tetC* and *qnsR* (Guo et al., 2017; Li et al., 2018; Choi et al., 2020; Su et al., 2020; Wang et al., 2021; Yang et al., 2021; Huang et al., 2022; Liang et al., 2022). To further illustrate the relationship between microbial composition, distribution of ARGs and geographical environment in waters of this region, the metagenomic next-generation sequencing (mNGS) was employed for analyzing microbiome and resistome in waterways around hospital, community, and natural wetlands in Wenzhou city. Besides the qPCR, the levels of key contaminants in the water samples, which were previously shown to be contributing to the distribution of ARGs, were also tested. This study will enhance our understanding of pathogenic bacteria, MDR bacteria, ARGs, and their abundance and distribution in urban water habitats.

## 2. Materials and methods

### 2.1. Sampling and collection

The workflow of the study was shown in Supplementary Figure S1. It was carried out in Wenzhou, an east coast city of Zhejiang province, China approximately at latitude 120°63′ E to 120° 74′E and longitude 27° 94’N to 28° 03’N. During April to June 2022, twenty water samples were collected from unique location, including 7 sites around hospitals (<20 m), 7 sites near community (<20 m), and 6 sites within natural wetlands. The distribution and flow direction of the waterways is shown in Supplementary Figure S2. After collection, water samples (2 L per sample) were transported to the laboratory on ice bags for analysis within 4 h. Each sample was divided into three fractions, 200 mL was sent for the detection of water quality index, 100 mL was used for microbial cultivation and identification, and 1,500 mL was taken for total DNA extraction.

### 2.2. Water quality index

Water samples were taken and characterized following National Water Quality Standard (NWQS) for the river in China in terms of pH, temperature, total nitrogen (kit equivalent to ISO 11905), total phosphorus (spectrophotometry, Standard Methods 4,500-P), and ammonia nitrogen (kit equivalent to ISO7150/1–1948).

### 2.3. Microbial cultivation and count

Briefly, 10 mL of water samples were homogenized in 0.1% peptone saline, and serial dilutions were cultured onto agar plates. Each sample was inoculated as triplicates on the target plate. Specifically, the total number of bacteria was determined by the plate counting method, antimicrobial-resistant bacteria were cultivated and numerated on Luria Bertani agar (AOBOX 01–001, Beijing, China) supplemented with tetracycline (20 μg/mL), sulfonamides (1,000 μg/mL), norfloxacin (20 μg/mL), erythromycin (10 μ g/mL), and penicillin (100 μg/mL). Strains of antimicrobial-resistant bacteria isolated were identified by MALDI-TOF MS (BioMérieux, Craponne, France). *Escherichia coli* were detected and primarily identified by Eosin-Methylene Blue Agar (AOBOX-EMBBR, Beijing, China). After incubation, suspected *E. coli* was identified using classical biochemical methods.

### 2.4. Metagenomic next-generation sequencing

Water samples were first centrifuged (11,200 g/min) and concentrated after being removed from the sampler. The concentrated water (250 μL) was used for metagenomic DNA extraction. The genomic DNA was purified with MagaBio Soil/Feces Genomic DNA Purification Kit (Hangzhou Bioer Technology, Hangzhou, China) and quantified with the NanoDrop ND-1000 (Thermo Fisher SCIENTIFIC, United States) using the A260/A28 ratio. Then extraction from each sample was diluted to approximately 5 ng/μL and stored at −20°C until metagenomic next-generation sequencing. The PCR products were subjected to 1% agarose gel electrophoresis and then sequenced on an Illumina (PE 300) platform of Honsunbio company (Shanghai, China).

### 2.5. Bioinformatics analysis

The pair-end reads for each sample were filtered for adapters and low-quality bases using the BBDuk2 module in the BBtools suite v38.94. Specifically, remove reads that contain more than a certain percentage (default 40%) of low-quality bases (quality value <15). Remove N bases to reach a certain percentage of reads (default value 5 bp). Remove Adapter sequences and sequences less than 15 bp in length. The sequencing data sizes were evaluated between 1076.68 MB to 2447.96 MB according to the samples.

Then, taxonomic profiling was performed on the remaining reads using SPARSE, which employed a pre-computed reference database based on bacterial genomes from the NCBI RefSeq database, by picking only one genome from each single-linkage cluster of <0.01 genetic distance. The database was accessed on March 12, 2023. Pathogenic bacteria were identified by downloading the database of PathogenFinder 1.1 and blasting locally to match the certain species with the default value (blastp, evalue ≤1e-5). The *intl1* gene was extracted from the metagenomic data set, and the local comparison was performed according to the reference gene WP_000845048.1. The phylogenetic tree of *intl1* was constructed by MEGA v11 software.

The ARGs and identification of ARG hosts were predicted in relation to the carriage of plasmids using ARGpore2 (Wu et al., 2022). Generally, the default similarity cutoff for ARGs filtration was set as 0.9, while the default alignment length cutoff for filtering ARGs lastal results was 0.6. The number of threads used for parallel computing was set as default *t* = 1. Violin and Heatmap plots were generated using a free online platform^1^ for data analysis and visualization with default settings.

Gene abundances associated with methane, nitrogen, phosphorus, and sulfur metabolism, as well as membrane transporters, were obtained by aligning against corresponding reference sequences in MCycDB, NCycDB, PCycDB, SCycDB, and TCDB, respectively, and normalized by their deviation to the average values for each category as $Odds=\left(d_{s}-\bar{d}\right)/\sigma$, where $d_{s}$ is the read depth per million reads in sample *s*, and $\bar{d}$ and σ are the mean and standard deviation of read depths for all samples. Default values were set as 0.7.

Finally, Humann3 v3.5 was used to estimate the abundances of other enzymes in metabolic pathways, based on the databases of nucleotide (full_chocophlan.v201901_v31), protein (uniref50_ec_filtered_201901b_subset), and utility_mapping (full_mapping_v201901b). Detailly, the minimum percentage of reads matching a species was set as default = 0.1. The query coverage threshold for nucleotide/translated alignments was 90.0, and subject coverage threshold for nucleotide/translated alignments was 50.0. Moreover, the value threshold to use with the translated search was employed as default = 1.0, while the index of the gene in the sequence annotation was set as default = 3.0. After that, genes that differed between groups were identified using a LEfSe test in^2^ and designated based on associated records in the BRENDA Enzyme Database.

### 2.6. Qualitative PCR validation

With the specific primers listed in Table 1, qPCR was employed to quantify the presence of intI1, *tetA, ermA, ermB, qnrB, sul1, sul2, bla*_SHV_, and *bla*_CTX-M_ in water samples. Reactions were conducted in 96-well plates with a final volume of 20 μL, including 10 μL iQ™ SYBR® Green Supper Mix (BioRad, Hercules, CA), plus 1 μL each primer (2 mM) and 8 μL template DNA. Thermal cycling and fluorescence detection were conducted on a BioRad iCycler with the software iCycler iQ version 3.0 (BioRad, Hercules, CA), using the following protocol: 94°C for 3 min, followed by 45 cycles of 94°C for 30 s, 50°C to 65°C for 30 s, 72°C for 60 s. Each reaction was run with a duplicate. Calibration curves (Ct value versus Log value of initial target gene copy number per reaction) with seven points for each qPCR were generated using tenfold serial dilutions of the plasmid-carrying target gene. Calibration curves were run together with each measurement. The PCR efficiencies ranged from 90.5 to 98.1%, and R 2 values were over 0.994 for all calibration curves. Based on the calibration curves, the Ct value of a test sample with an unknown concentration was used to calculate copy number of target genes, and then the latter was normalized against the mass (ng) of the extracted DNA and the volume (mL) of original samples.

**Table 1:** Primers for qualitative PCR.

Gene	Forward primer (5′ → 3′)	Reverse primer (5′ → 3′)	Amplification length (bp)	Annealing temperature (°C)
16S rRNA	CGGTGAATACGTTCYCGG	GGWTACCTTGTTACGACTT	142	60
*intI*1	CCTCCCGCACGATGATC	TCCACGCATCGTCAGGC	280	55
*tetA*	CTCACCAGCCTGACCTCGAT	CACGTTGTTATAGAAGCCGCATAG	71	59
*ermA*	TTGAGAAGGGATTTGCGAAAAG	ATATCCATCTCCACCATTAATA GTAAACC	76	57
*ermB*	AGCCATGCGTCTGACATCTA	CTGTGGTATGGCGGGTAAGT	193	55
*qnrB*	TCGGCTGTCAGTTCTATGATCG	TCCATGAGCAACGATGCCT	496	54
*sul*1	CGCACCGGAAACATCGCTGCAC	TGAAGTTCCGCCGCAAGGCTCG	162	65
*sul*2	TCATCTGCCAAACTCGTCGTTA	GTCAAAGAACGCCGCAATGT	105	59.5
*bla*_SHV_	GCGAAAGCCAGCTGTCGGGC	ATTGGCGGCGCTGTTATCGC	303	61
*bla*_CTX-M_	ATGTGCAGYACCAGTAARGT	TGGGTRAARTARGTSACCAGA	593	50

### 2.7. Statistical analysis

The analysis of diversity, variance, and similarity with repeated measures was used to determine the statistical significance for each experimental group. The one-way analysis of variance (ANOVA) was conducted using GraphPad Prism (v7) and Tutools platform with statistical significance accepted as *p < 0.05, **p < 0.01, ***p < 0.005, ****p < 0.001. A Spearman’s correlation was used to examine the relationship between the water quality index and types of ARGs in this study.

## 3. Results

### 3.1. Water samples from hospital surrounds showed higher concentrations of nitrogen, phosphate, and greater numbers of antimicrobial-resistant bacteria

**Figure 1:**
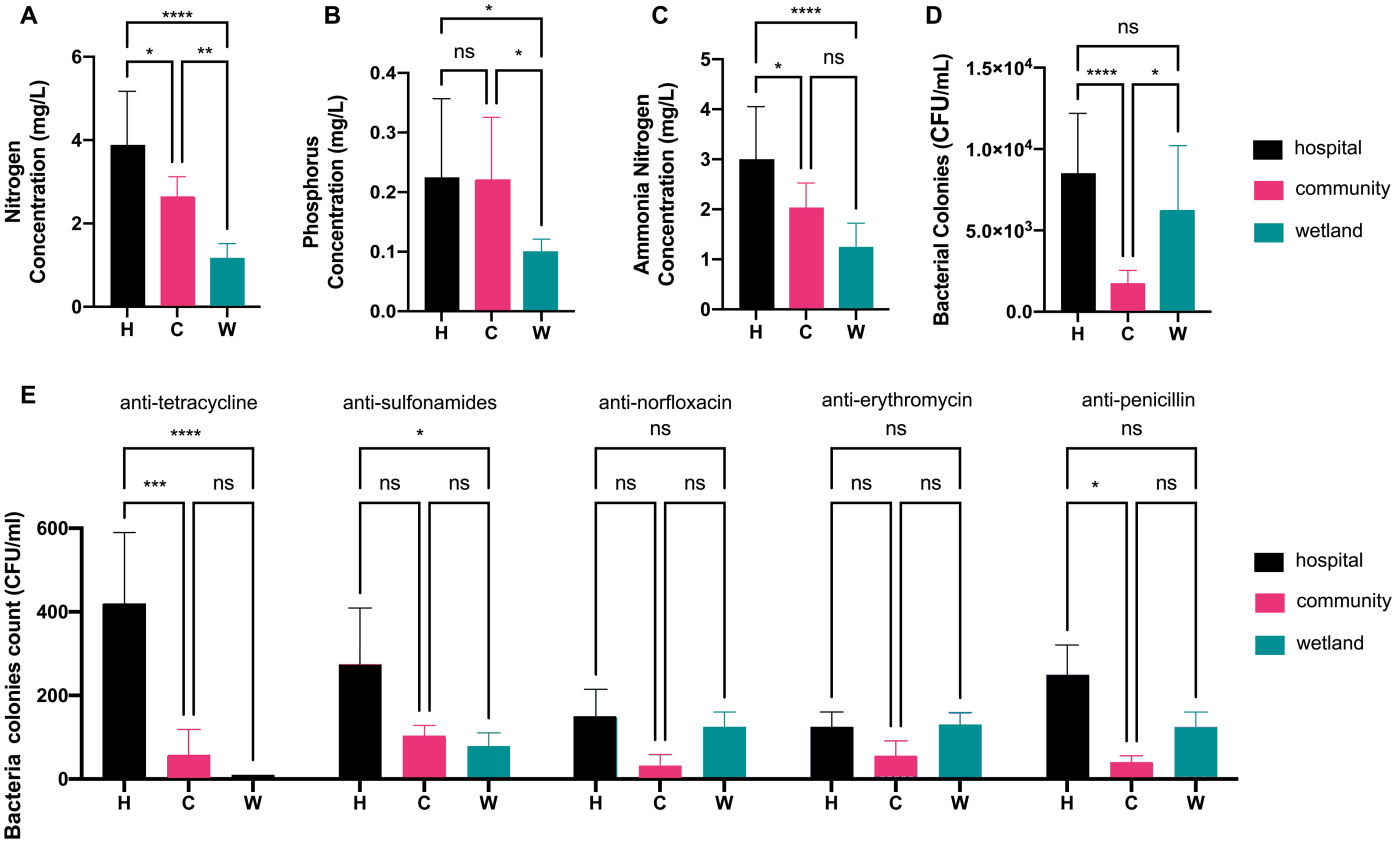
Analysis of water quality index and colonies counts of antimicrobial-resistant bacteria. Barplot showed the concentrations of **(A)** nitrogen, **(B)** phosphorus, and **(C)** ammonia nitrogen in hospital, community, and wetland water samples, respectively. **(D)** The colonies counts of total mesophilic aerobic bacteria. **(E)** The colonies counts of bacterial strains resistant to tetracycline, sulfonamides, norfloxacin, erythromycin, and penicillin. *p < 0.05, **p < 0.01, ***p < 0.005, ****p < 0.001.

This study took water samples from urban waterways near the hospital (H; *n* = 7), community (C; *n* = 7), and wetlands (W; *n* = 6) in Wenzhou from April to June 2022 (Supplementary Figure S2). All sampling sites shared the same temperature of 22°C and overall pH of 7.6. The groups differed from each other by their chemical components and bacterial loads. The indexes of total nitrogen, phosphorus, and ammonia nitrogen in the water around hospital were 2 to 3-fold as high as that of water from wetland, while the water around the community always had medium levels of chemicals (Figures 1A–C). In contrast, the water around hospital and wetland shared similar levels of bacterial counts of 3.90 ± 0.17 and 3.78 ± 0.15 log CFU/mL, while the bacterial loads in water around communities were 6 to 8-fold fewer (Figure 1D). The numbers of bacterial colonies that grew in antimicrobial agents were also different. The water around hospitals carried greater amount of strains resistant to tetracycline and sulfonamides than those from communities and wetlands. In contrast, the amount of strains resistant to norfloxacin, erythromycin and penicillin were not significantly different between water around hospitals and those from the others (Figure 1E).

### 3.2. Variations in microbial abundance and diversity from different urban water habitats

**Figure 2:**
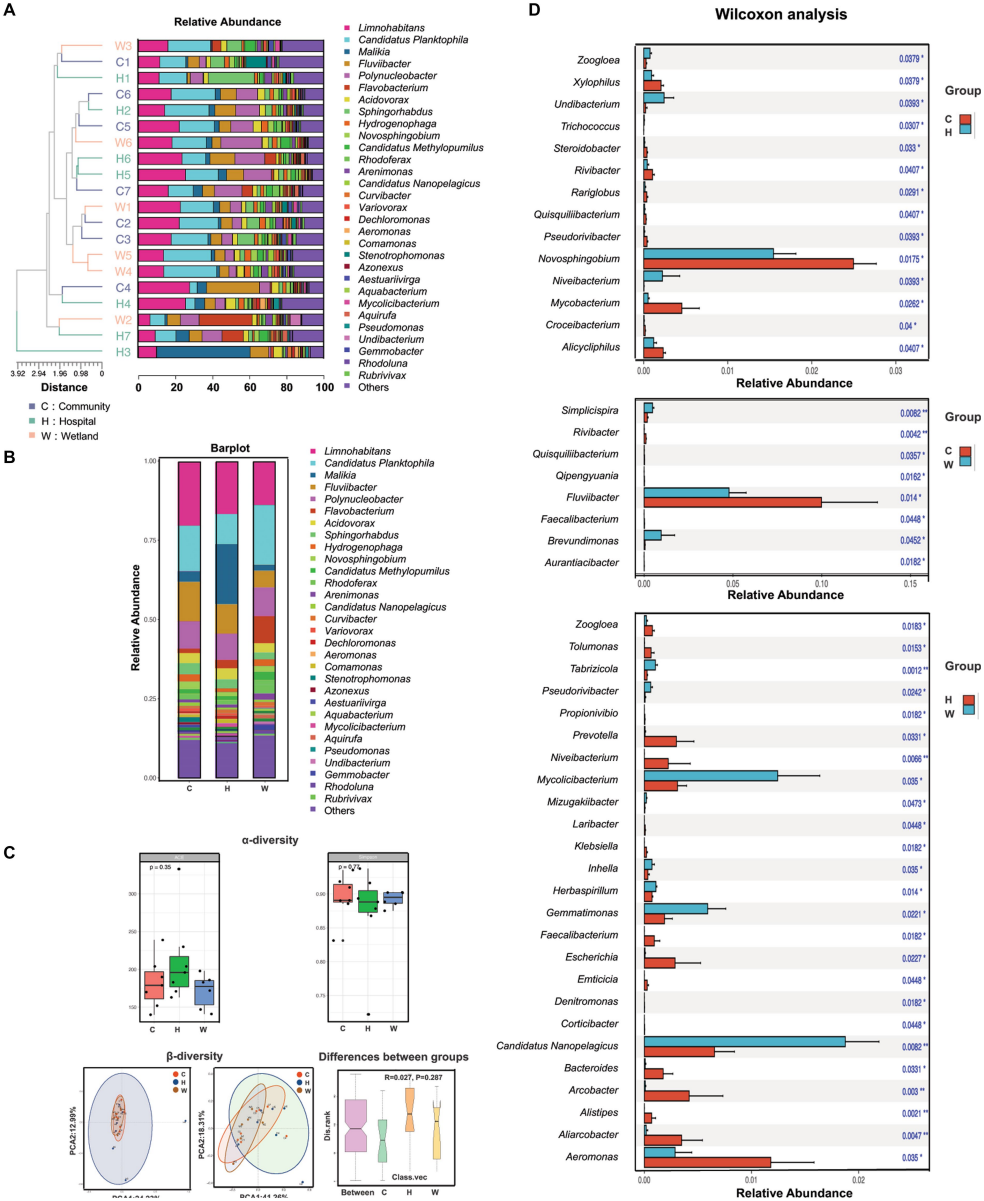
The composition, abundance and diversity of bacterial genera in water samples collected from hospitals, communities, and wetlands, respectively. **(A)** Characteristics of bacterial genus. The phylogenetic tree on the left was the genetic distance of bacteria in each water source. Different color blocks in the histogram represented bacterial genera. **(B)** Characteristics of bacterial species. Bar graph showed the overall bacterial distribution in the three water bodies. Different color blocks in the histogram represented bacterial genera. **(C)** Analysis of α-diversity and β-diversity in three water bodies and the variations between groups. **(D)** Wilcoxon analysis showed the bacterial genera with significant abundance in pairwise comparisons of each group (C vs. H, C vs. W, H vs. W).

A total of 1,594 bacterial species from 479 genera were identified from the water samples. More than half (246/479) of the identified genera were presented in all three groups, while only 165 genera were uniquely present in one group. The hospital-related samples had the greatest number of unique genera (120), followed by those from wetlands (25) and communities (20). This increased taxonomic richness was largely associated with the greater inter-sample diversity (beta-diversity) among samples surrounding hospitals compared to the other two groups, rather than the alpha-diversity within each sample, where the hospital-related samples had slightly lower Shannon and Simpson indexes than the others (Figures 2A–C). However, all these differences led to only a minor shift in the overall taxonomic compositions in the hospital-related samples. The samples intermixed together in the UPGMA clustering (Figure 2B) and the PCA plots (Figure 2C).

We also evaluated the genera that differed between the groups. The genera that were mostly enriched in the community-related samples were *Limnohabitans*, *Fluviibacter* and *Novosphingobium* (Figure 2D), which are associated with enhanced degradation of contaminants, especially aromatic compounds. The hospital-related samples contained a large number of bacteria associated with the gut microbiome, including *Alistipes*, *Prevotella*, *Klebsiella*, *Escherichia*, *Bacteroides*, and *Faecalibacterium*, which were all significantly enriched compared to samples from the wetlands. The wetland waters had enriched bacteria from *Nanopelagicus*, *Mycolicibacterium* and *Gemmatimonas*, which are typically associated with aquatic environments.

### 3.3. Antimicrobial resistance genes were enriched in urban water habitats together with mobile genetic elements

**Figure 3:**
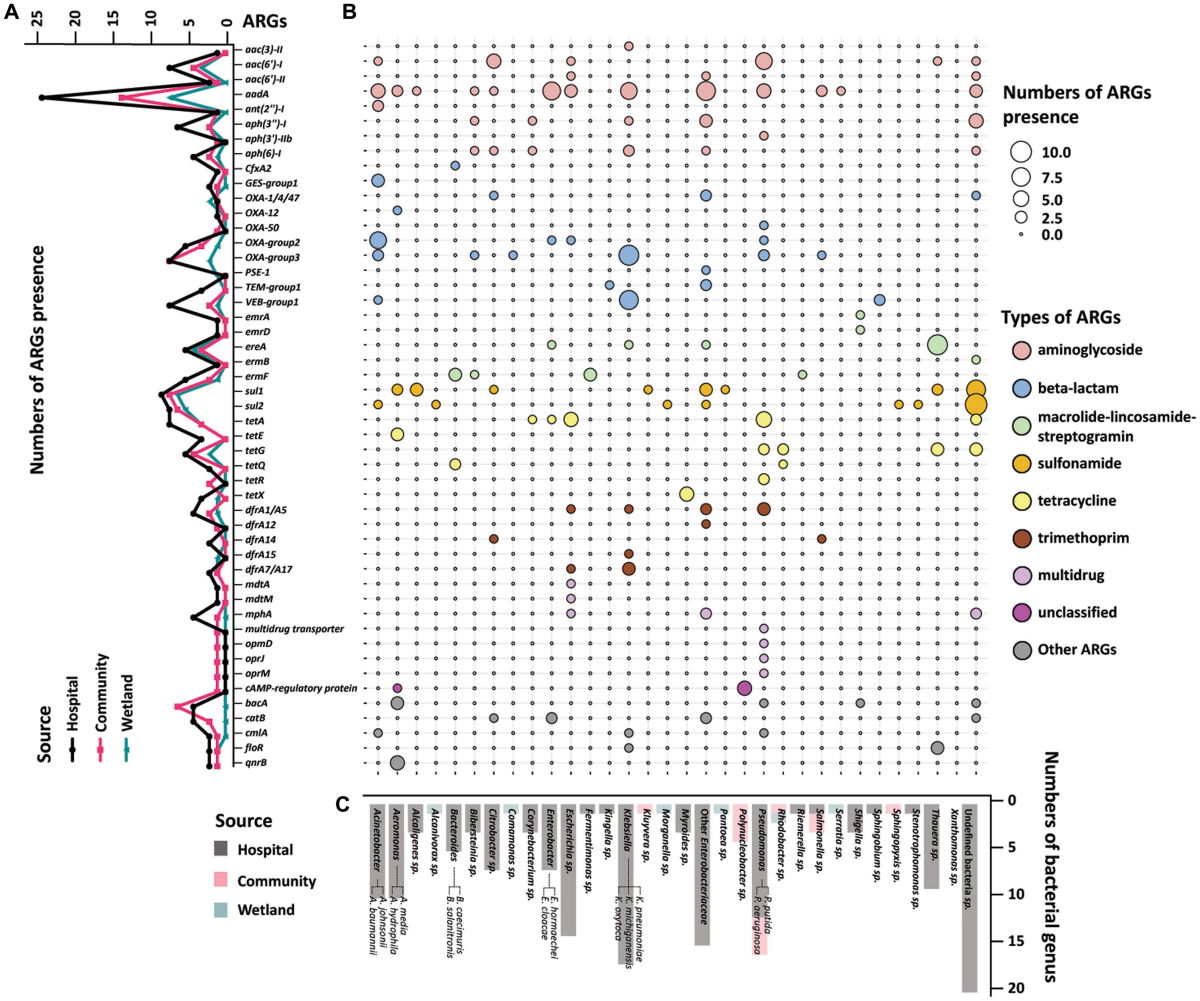
Profile of resistome of water samples from hospital, community, and wetland. **(A)** The line graph on the left showed the accumulation of ARGs according to the mNGS of the three water bodies. The X-axis showed various ARGs, and the Y-axis was the number of ARGs detected in the water samples. The black line represented the accumulation of ARGs in rivers around hospitals, and red and green lines represent the ARGs near communities and wetlands. **(B)** Heatmap indicated the accumulation of ARGs in water samples in different bacterial genera. The size of the circle represented the numbers of the accumulated ARGs. The larger circles indicated the higher amount of ARGs in this genus. The colors of the circles represented different classes of antimicrobials. **(C)** The histogram on the bottom side showed the distribution of strains carrying ARGs among the three water bodies. The X-axis showed different genus of bacteria, and the Y-axis was the number of the bacteria detected in the metagenomics. The black bars were the distribution of bacteria in the hospital water, and the red and green bars were the distribution of bacteria in the community and wetlands.

**Figure 4:**
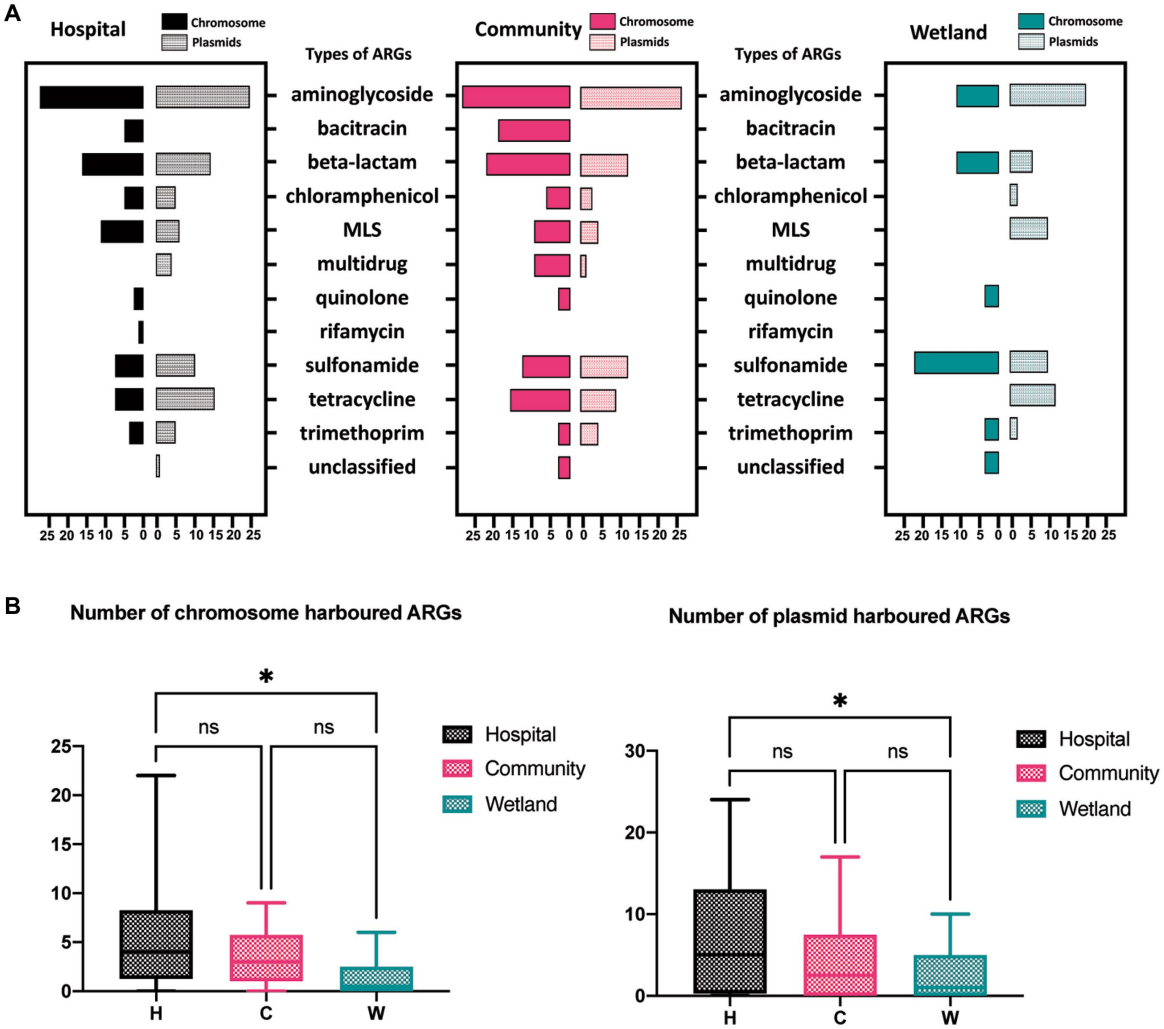
The distribution of ARGs carried by plasmids, and chromosomes in water samples. **(A)**The barplot showed the overall distribution between hospital, community, and wetland environments. The numbers at the bottom represent the numbers of ARGs carried on chromosomes and plasmids, respectively. **(B)** Analysis of the difference in the number of ARGs carried by chromosomes and plasmids indicated that the number of ARGs in the water around the hospital was significantly higher than that in the wetland group. *p < 0.05, **p < 0.01, ***p < 0.005, ****p < 0.001.

The presence of ARGs that were associated with different species origins in each sample was estimated using ARGpore. The greatest amount of ARGs were found in samples around hospitals, followed by samples around communities and wetlands (Figure 3A). The majority of ARGs from hospital-related samples were carried by bacteria from *Acinetobacter*, *Aeromonas* and various genera from *Enterobacteriaceae*, which each was associated with >5 ARGs (Figures 3B,C). In contrast, the ARGs that were exclusively in samples from communities and wetlands were carried by species that encoded only 1–2 ARGs each and were not normally associated with human infections. We also investigated the relationship between ARGs and mobile elements in water samples (Figure 4). Intriguingly, the type and quantity of plasmids in wetlands water were significantly lower than those in hospitals and communities (Figure 4B, *p* < 0.05). The *intI1* gene was detected in 15 water samples, with majority of them sharing similar genomes (Supplementary Figure S3). The hospital water contained a considerable number of opportunistic pathogenic bacteria and ARGs such as *bla*_OXA_*, bla*_TEM_*, bla*_VEB_*, and* other genes. Notably, aminoglycosides, β-lactams, and tetracyclines were the top three antimicrobials currently associated with the horizontal transferring of plasmids in water samples (Figure 4A).

### 3.4. Qualitative PCR validated the presence of ARGs in urban aquatic habitats

**Figure 5:**
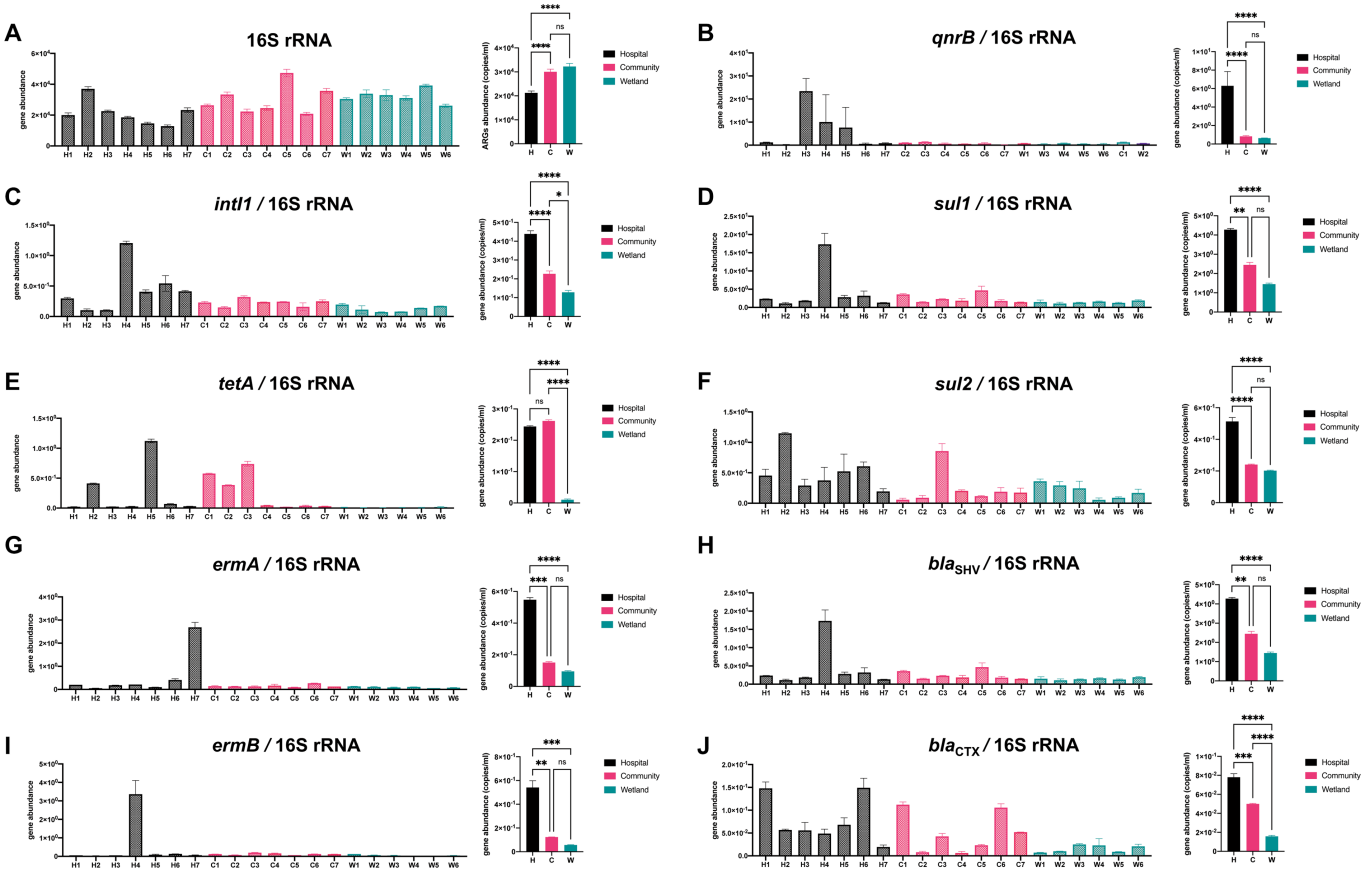
Qualitative PCR was used to detect presence of ARGs in urban aquatic habitats. A total of 12 genes were analyzed including **(A)** 16S rRNA, **(B)** *qnrB*, **(C)** *intI1*, **(D)** *sul1*, **(E)** *tetA*, **(F)** *sul2*, **(G)** *ermA*, **(H)** *bla*_SHV_, **(I)** *ermB*, **(J)** *bla*_CTX_. Both *intI1* and ARGs were normalized to the 16 s results. The one-way analysis of variance (ANOVA) was enrolled for calculation with *p < 0.05, ** *p* < 0.01, *** *p* < 0.005, **** *p* < 0.001.

The abundances of 10 genes (*tetA, ermA, ermB, qnrB, sul1, sul2, bla*_SHV_, *bla*_CTX-M_, *intI1*, and 16S rDNA, Figure 5) were quantified using qPCR. There was a significant variation in the abundance of 16S rDNA between samples from different groups, with hospital-related samples containing one-third fewer copies. The abundance of the *intI1* gene varied from 4.25 × 10^2^ copies/mL in hospital-related samples to 1.10 × 10^4^ copies/mL in the wetlands (Figure 5). Furthermore, *tetA* was substantially more prevalent in hospital and community settings than in wetlands. Compared to the other two groups, hospital ambient water had considerably greater *ermA*, *qnrB,* and *sul2* gene abundance, while the community region had the highest abundance of *bla*_SHV_ (Figure 5).

### 3.5. The metabolism of the water microbiome is associated with surrounding environment

**Figure 6:**
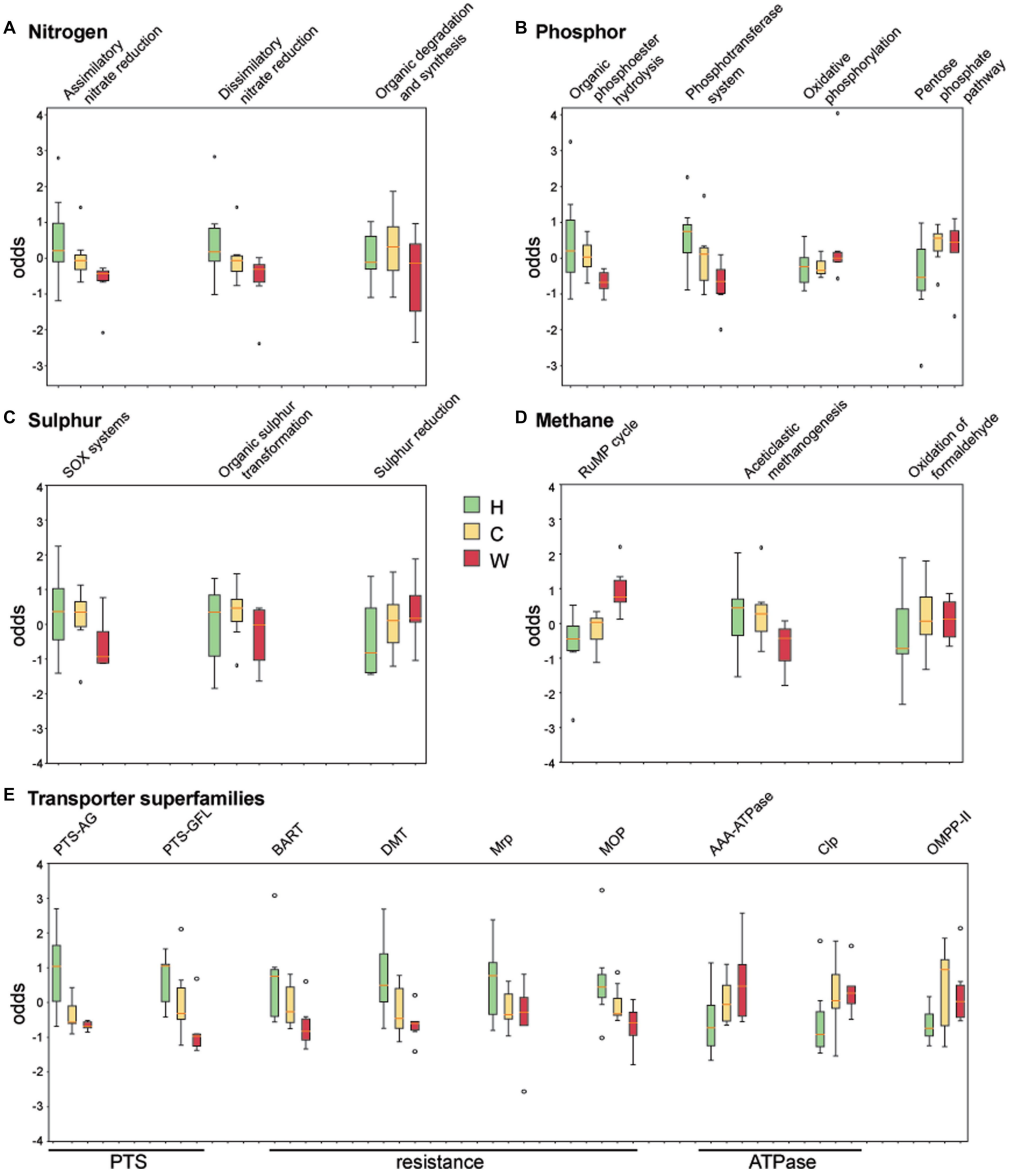
Analysis of chemical index and enzyme metabolic pathways. The coverage of genes associated with **(A)** nitrogen, **(B)** phosphor, **(C)** sulphur, **(D)** methane, and **(E)** transporter superfamilies were obtained by comparing against curated datasets in NcycDB, PcycDB, ScycDB, McycDB and TCDB. Sulfur oxides (SOx), ribulose monophosphate (RuMP), phosphotransferase system (PTS), phosphotransferases system (PTS-AG), phosphotransferases system (PTS-GFL), Bile/Arsenite/Riboflavin Transporter (BART), drug/metabolite transporter (DMT), Na + Transporting Mrp (Mrp), multidrug/oligosaccharidyl-lipid/polysaccharide (MOP), ATPases Associated with Diverse Cellular Activity (AAA ATPase), ATP-dependent Clp Protease (Clp), Outer Membrane Pore-forming Protein II (OMPP-II).

The coverage of genes associated with nitrogen, phosphor, sulphur, methane cycling, and transporters was obtained by comparing against curated datasets in NcycDB, PcycDB, ScycDB, McycDB and TCDB, respectively. Functional pathways or superfamilies that varied across environments are shown in Figure 6. Genes associated with the degradation/utilization of nitrate and organic phosphodiester were enriched in water samples around hospitals and communities compared to those from wetlands (Figures 6A,B), possibly due to the higher indexes of total nitrogen and phosphorus in these samples. In addition, water around hospitals showed an enrichment of genes associated with sulfur oxidation, the phosphotransferase system (PTS), and transporters associated with drug resistance (Figures 6B,C,E), likely associated with resistance to antimicrobials and/or chemicals. Genes associated with oxidative phosphorylation, methane oxidation, and ATPases (Figures 6B,D,E) were enriched in water from wetlands, suggesting a higher level of aerobic respiration compared to the other two groups, and possibly associated with its greater level of protein synthesis GTPase (EC 3.6.5.3). Finally, the water around communities was enriched in EC 1.3.8.1, which is short-chain acyl-CoA dehydrogenase, a crucial enzyme in fatty acid oxidation.

### 3.6. Relations between the presence of water quality indicators, ARGs and geographical environment

We compared the correlations between the number of ARGs and water quality indicators (Table 2). The contents of total nitrogen and total phosphorus were significantly correlated with the presence of *ermB* (*p* = 0.0444*) and *sul1* (*p* = 0.0398*), respectively. Besides, ammonia nitrogen was also associated with *intI1* and *sul1* (*p* = 0.0337* and *p* = 0.0458*). Furthermore, *intI1* exhibited a significant correlation with *ermB* (*p* = 0.0021**), *sul1* (*p* < 0.0001***), and *bla*_SHV_ (*p* = 0.0458*), indicating a prevalence of ARGs in urban water environments due to the integron *intI1*’s diffusion-promoting effect. The remaining ARGs were not significantly correlated to water quality indicators. In addition, the high abundance of ARGs was limited to the waters around the hospital, and we did not observe the geographical transfer of ARGs along with the river flow. For example, the rivers from No.7 hospital and No.1 hospital showed great abundance of *bla*_OXA_*, ermA, sul* and other ARGs (Supplementary Figure S2), while it was not the case for waters from No.4 and No.5 wetlands in the downstream of the waterways (Supplementary Figure S2). Nevertheless, a similar microbial composition and ARGs modes were detected with specific regions, e.g., No. 2 hospital and No. 6 community, No. 1 wetland and No.2/3 community (Figure 5, and black dotted circle in Supplementary Figure S2), indicating that the presence of certain ARGs was restricted to the water bodies near the hospital, without significant transmission along the river flow.

**Table 2:** Correlations between ARGs and water quality index.

Variable^#^	Total nitrogen	Total phosphorus	Ammonia nitrogen	*intI1*	16S rRNA
16S rRNA	−0.0244	0.2457	−0.1522	/	/
P16S rRNA	0.9101	0.2472	0.4778	/	/
*intI1*	0.2896	0.3405	0.4348	/	/
P*intI1*	0.1699	0.1035	0.0337*	/	/
*tetA*	0.0774	0.3053	0.1226	0.3774	0.247
P*tetA*	0.7193	0.1469	0.5682	0.0691	0.2447
*ermA*	−0.3174	−0.0761	−0.1809	−0.2096	−0.4983
P*ermA*	0.1307	0.7238	0.3977	0.3257	0.0132*
*ermB*	0.4139	0.1666	0.3696	0.5957	−0.247
P*ermB*	0.0444*	0.4367	0.0755	0.0021**	0.2447
*qnrB*	0.04	−0.0048	0.0539	−0.0365	−0.1148
P*qnrB*	0.8528	0.9823	0.8024	0.8655	0.5933
*sul1*	0.2574	0.4222	0.4113	0.7878	0.1444
P*sul1*	0.2247	0.0398*	0.0458*	<0.0001**	0.501
*sul2*	0.0548	0.1914	0.167	0.347	0.4600
P*sul2*	0.7993	0.3704	0.4355	0.0967	0.0237*
*bla*_SHV_	−0.0496	0.3884	0.1896	0.4113	0.2991
P*bla*_SHV_	0.8181	0.0608	0.375	0.0458*	0.1556
*bla*_CTX-M_	−0.0678	−0.2461	−0.3444	0.0809	0.2391
P*bla*_CTX-M_	0.7528	0.2463	0.0994	0.7072	0.2604

^#^tetA and PtetA represent the correlation coefficient and value of *p* of the corresponding ARGs and water quality index, respectively. Other variable genes were the same. ^*^p < 0.05, ^**^p < 0.01, ^***^p < 0.005, ^****^p < 0.001.

## 4. Discussion

The presence of pathogenic bacteria in water environments is of a growing concern, as surface water is the source of drinking water, livestock feeding, and agricultural irrigation within the hydrologic cycle (Nnadozie and Odume, 2019). The high levels of total nitrogen and phosphors suggested that the water body near the sample location was at risk of eutrophication, which leads to enrichment of associated microbes, and raises the risk of disease transmission and the spread of antimicrobial resistance genes (Holmes et al., 2016; Hao et al., 2019).

We spot a strong association between *Enterobacterieae* and the enrichment of extended-spectrum β-lactamases and other ARGs in water from hospital surrounds, which differed from waters in wetlands where only 1–2 ARGs were carried by each species. This finding is consistent with previous studies that have reported frequent isolations of ESBL-carrying *E. coli* from riverine and surface water environments around hospitals (Dirar et al., 2020; Lü et al., 2020). The global dissemination of the *bla*_CTX-M_ gene has been reported in hospital settings, communities, livestock, and companion animals (Jamborova et al., 2018). In addition, the *bla*_CTX-M-27_ containing isolates have also been widely reported in other Asian countries, including Japanese and Korean hospitals, suggesting regional transmission of these ESBL genes (Miyagi and Hirai, 2019; Kim et al., 2021).

Another interesting finding in this study was the correlation between presence of *intI1* and the emergence of ARGs. Previous studies have shown that the integron *intI1* facilitated the horizontal transfer of antimicrobial resistance genes (Kayali and Icgen, 2021). The association between *intI1* and MDR strains had been extensively reported. For example, Ejaz et al. found correlations of *bla*_TEM_, *bla*_SHV_, and *bla*_CTX-M_ in clinical isolates of *intI1* (Ejaz et al., 2021). Li et al. reported potential interspecies transfer of ARGs mediated by *intI1*, suggesting the integron promoting effect (Li H. et al., 2021). Together, these findings highlight the critical role of the *intI1* in the dissemination of antimicrobial resistance genes among human and environmental bacteria.

Notably, despite the varying levels of contaminants and the enrichment of human pathogens carrying ARGs in urban environments, the water from all sources shared a large proportion of their microbiome (An et al., 2023). The water samples from the geographically related sites, such as No. 4 W/No. 5 W, and No. 5H/No. 6H (Supplementary Figure S2), exhibited similar taxonomic profiles independent of the close geographical distance. Similar observations had been reported in previous studies that water bodies had a certain self-purification capacity, which allows ARGs to be dissolved without spreading far along the river (Maurya and Raj, 2020). Although similar total bacterial compositions were found in some geographically closely located regions, the bacteria carrying most of ARGs, i.e., *Acinetobacter* and *Enterobactereae*, as well as the corresponding vectors (*IntI*) were found to be significantly higher in water bodies near the hospital than the other two water settings (Figure 5).

The relations between the presence of water quality indicators and ARGs were evaluated in this study. The indicators of total nitrogen, phosphorus, and ammonia nitrogen were significantly correlated with the presence of *ermA* and *sul1*. However, no significance was observed among the remaining ARGs. Previous studies had revealed that inorganic nutrients (ammonia, nitrate, nitrite and phosphorus) played important roles in promoting ARG transfer in the water (Wang et al., 2021; Yang et al., 2021; Huang et al., 2022). Yang et al. reported that the concentrations of ammonium-nitrogen (NH4 + -N), and phosphorus in pig farms were significantly correlated with six types of ARGs including genes resistant to tetracyclines, sulfonamides, beta-lactams and aminoglycosides (Yang et al., 2021). Besides, a study by Wang et al. showed that water quality indicators in the river were positively correlated with the abundances of *sul1, sul2, tetA, tetB, tetC,* and *qnsR* (Wang et al., 2021). A number of studies had evidenced that the occurrence and spread of ARGs in water environments could be determined by various biotic and abiotic driving factors, such as total nitrate and total phosphorus. This may be due to the enrichment of genes related to bacterial sulfur oxidation and phosphotransferase systems (PTS) in the hospital environment, which are further transcribed and expressed as manifestations of bacterial resistance to antimicrobials and/or chemicals (Guo et al., 2017; Li et al., 2018; Choi et al., 2020; Su et al., 2020; Wang et al., 2021; Yang et al., 2021; Huang et al., 2022).

Notably, Fungi are also an important part of environmental microorganisms, which have a significant impact on the abundance and diversity of ARGs. However, our metagenomic DNA extraction approach was only applicable to prokaryotes and not fungi or other eukaryotes. For the time being, our data cannot be used to assess the composition of fungus in water. Further research is needed to elucidate the association between water quality indicators and the distribution of antimicrobial resistance genes in water habitats.

Similar patterns were observed in metabolic pathways, where genes associated with degradation of contaminants were enriched in water with high levels of corresponding chemicals. Nevertheless, compared to water from wetlands, the urban water showed reduced aerobic respiration and increased methane production, which could lead to a systematic shift of the microbiome towards anaerobic respiratory in the long term (Griebler et al., 2016). For example, we observed an enrichment of short-chain acyl-CoA dehydrogenase around communities. This was a crucial enzyme in fatty acid oxidation that speeded fatty acid conversion and improves the metabolic pathways of carbon dioxide nutrition and acetate nutrition (Messina et al., 2020). Other studies also indicated that the gene expression of such metabolic enzymes increased during the process of lignite anaerobic fermentation to create methane (Duan et al., 2021; Gao et al., 2021).

In summary, our findings demonstrated that water samples collected around hospitals exhibited elevated concentrations of nitrogen and phosphate, indicating a greater enrichment with nutrients. Both metagenomes and qualitative PCR validated the presence of clinical pathogenic bacteria in hospital water samples, along with higher microbial abundance and diversity. Furthermore, we identified multiple ARGs in the current urban river, with aminoglycoside, beta-lactam, and tetracycline resistance being the most prevalent. The mobilization of these ARGs may be facilitated by plasmids and the *intI1* integron. However, the high abundance of ARGs was limited to the waters around the hospital, and we did not observe the geographical transfer of ARGs along with the river flow. This may be related to water purifying capacity of natural riverine wetlands (Sileshi et al., 2020). Continued surveillance is required to assess the risk of bacterial horizontal transmission and its potential impact on public health.

## Data availability statement

The datasets presented in this study can be found in online repositories. The names of the repository/repositories and accession number(s) can be found at: https://ngdc.cncb.ac.cn/gsa/browse/cra009676, cra009676.

## Author contributions

YL, ZZ, and JD conceived and supervised the project and designed the experiments. SheY contributed to the design of the experiments and performed most of the experiments. CS, HL, and SL analyzed the sequencing results and wrote the manuscript.WZ, SD, and SheY collected the water samples. ZS, SW, JH, JZ, and ShuY performed parts of the experiments. SheY, HL, and SL analyzed the data and drew the figures. ZZ and JD revised the manuscript. All authors contributed to the article and approved the submitted version.

## Funding

This work was supported by Zhejiang Provincial Natural Science Foundation of China (Grant no. LGF18C050002) and Zhejiang Medical and Health Science and Technology Project (Grant no. 2018PY030).

## Conflict of interest

The authors declare that the research was conducted in the absence of any commercial or financial relationships that could be construed as a potential conflict of interest.

## Publisher’s note

All claims expressed in this article are solely those of the authors and do not necessarily represent those of their affiliated organizations, or those of the publisher, the editors and the reviewers. Any product that may be evaluated in this article, or claim that may be made by its manufacturer, is not guaranteed or endorsed by the publisher.

## Footnotes

^1^ http://www.bioinformatics.com.cn

^2^ http://huttenhower.sph.harvard.edu/galaxy/

## Supplementary material

SUPPLEMENTARY FIGURE S1 Study workflow. Combined laboratory and bioinformatics analysis were used to illustrate the characteristics of water quality index, microbial composition and antimicrobial resistance genes in urban water habitats in Wenzhou city, Southeast China.

SUPPLEMENTARY FIGURE S2 Sampling map. Water samples were collected from 20 rivers, including seven from hospital surrounds, seven near community, and six from natural wetlands between April to June 2022. The distribution and flow direction of the rivers is shown in Figure. Triplicate samples were taken from one river.

SUPPLEMENTARY FIGURE S3 The phylogenetic tree of intl1. The intl1 gene was extracted from the metagenomic data set, and the local comparison was performed according to the reference gene WP_000845048.1.
