# Supplementary material for: Characterization of microbial community and antibiotic resistome in intra urban water, Wenzhou China
Source: Front Microbiol. 2023 Jun 15;14:1169476. doi: 10.3389/fmicb.2023.1169476 (PMC10311006; doi:10.3389/fmicb.2023.1169476)
Supplement: SUPPLEMENTARY FIGURE S1 — Study workflow. Combined laboratory and bioinformatics analysis were used to illustrate the characteristics of water quality index, microbial composition and antimicrobial resistance genes in urban water habitats in Wenzhou city, Southeast China. [file Image_1.pdf]

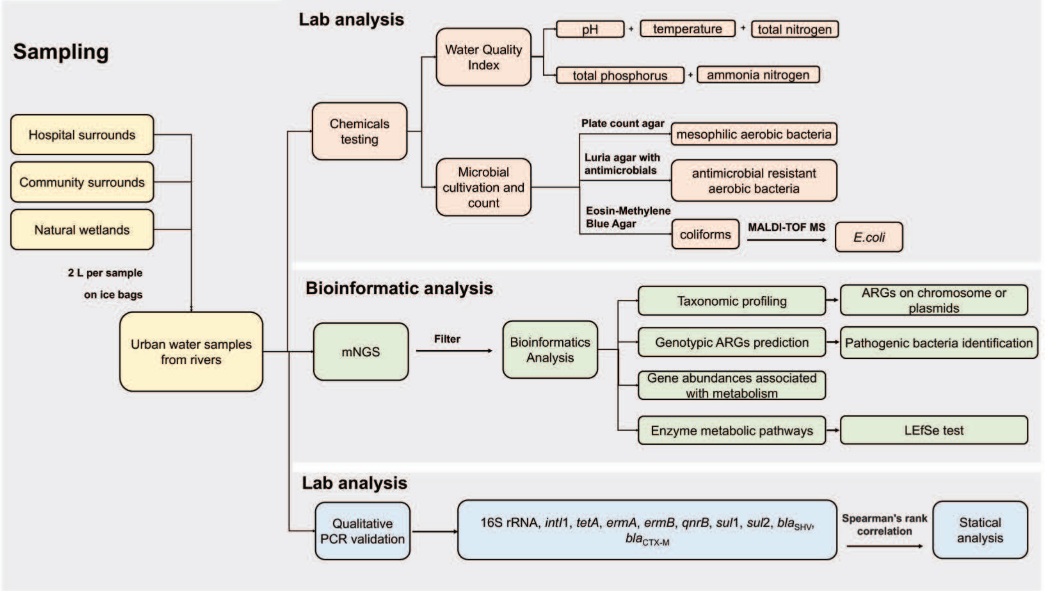
Sampling
Hospital surrounds
Community surrounds
Natural wetlands
2 L per sample
on ice bags
Urban water samples from rivers
Lab analysis
Chemicals testing
Water Quality Index
pH
temperature
total nitrogen
total phosphorus
ammonia nitrogen
Microbial cultivation and count
Plate count agar
mesophilic aerobic bacteria
Luria agar with antimicrobials
antimicrobial resistant aerobic bacteria
Eosin-Methylene Blue Agar
coliforms
MALDI-TOF MS
E.coli
Bioinformatic analysis
mNGS
Filter
Bioinformatics Analysis
Taxonomic profiling
ARGs on chromosome or plasmids
Genotypic ARGs prediction
Pathogenic bacteria identification
Gene abundances associated with metabolism
Enzyme metabolic pathways
LEfSe test
Lab analysis
Qualitative PCR validation
16S rRNA, intI1, tetA, ermA, ermB, qnrB, sul1, sul2, blaSHV, blaCTX-M
Spearman's rank correlation
Statical analysis

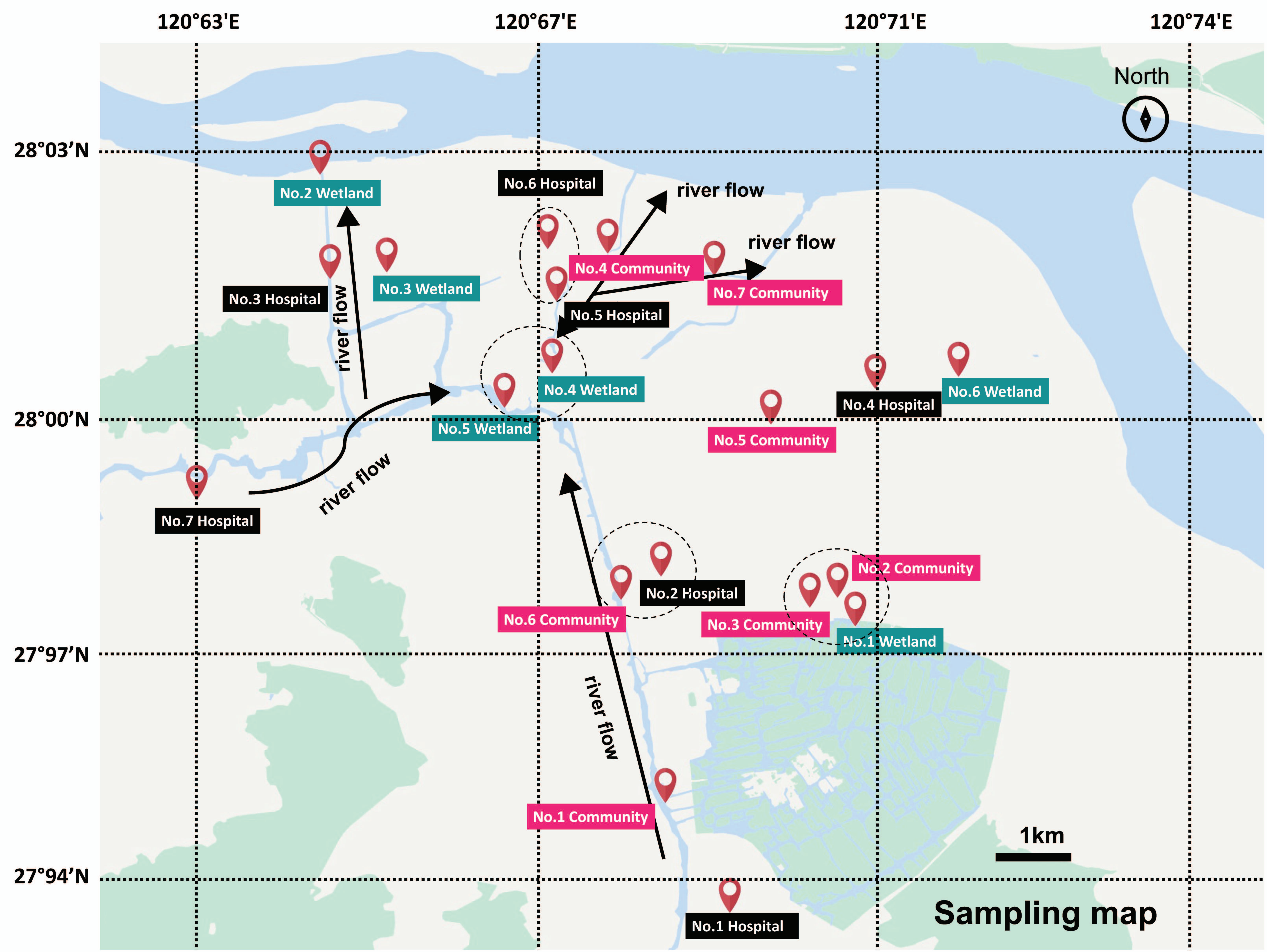
120°63'E
120°67'E
120°71'E
120°74'E
28°03'N
28°00'N
27°97'N
27°94'N
North
No.2 Wetland
No.6 Hospital
river flow
river flow
No.3 Hospital
No.3 Wetland
No.4 Community
No.7 Community
No.5 Hospital
river flow
No.4 Wetland
No.5 Wetland
No.6 Wetland
No.4 Hospital
No.5 Community
No.7 Hospital
river flow
river flow
No.2 Community
No.2 Hospital
No.6 Community
No.3 Community
No.1 Wetland
No.1 Community
1km
No.1 Hospital
Sampling map

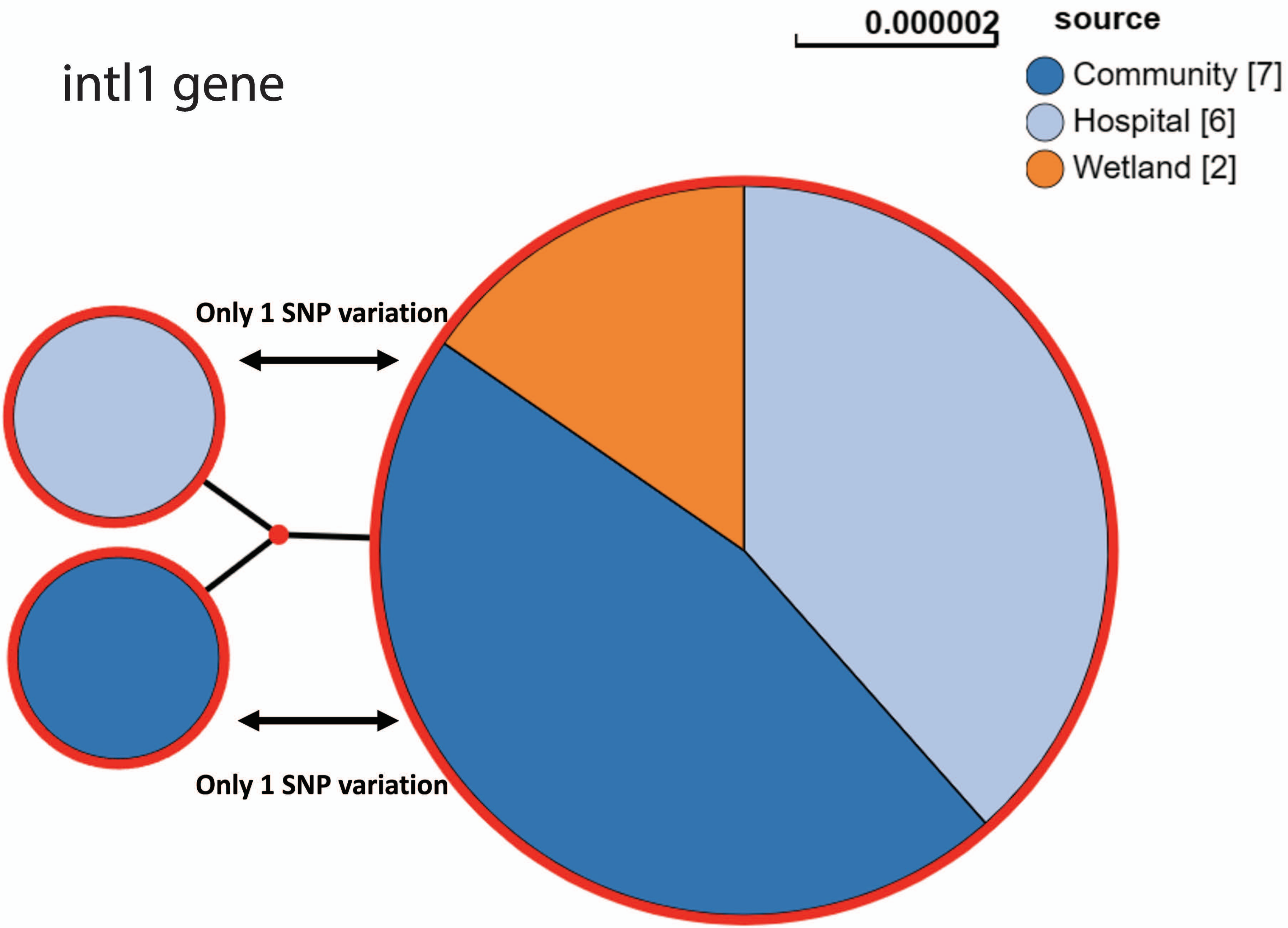

intl1 gene
0.000002
source
Community [7]
Hospital [6]
Wetland [2]
Only 1 SNP variation
Only 1 SNP variation
